# Caring by default: experiences of caregivers of children with developmental disabilities in Ghana mirrored in the context of the stress process model

**DOI:** 10.1186/s12912-024-02142-1

**Published:** 2024-07-15

**Authors:** Doreen Asantewa Abeasi, Nokuthula Gloria Nkosi, Ebenezer Badoe, Josephine Adjeman

**Affiliations:** 1 Department of Nursing Science Education, University of Witwatersrand, Johannesburg, South Africa; 2 Department of Nursing and Midwifery, Presbyterian University, Abetifi, Ghana; 3 Department of Child Health, Korle-Bu Teaching Hospital, Accra, Ghana; 4 University of Ghana Medical School, Korle-Bu, Accra, Ghana

**Keywords:** Caregivers, Children, Developmental disabilities, Experiences, Ghana

## Abstract

**Background:**

Caring for a child with developmental disabilities (DD) is associated with significant stress and burden. Caregivers’ experiences are influenced by factors such as poverty, stigma, and the lack of accessibility to services, equipment, and assistive devices. These factors are prevalent in a low-resource setting like Ghana which ultimately influences the experiences of caregivers. The aim of the study was to explore the experiences of caregivers of children with DD in the context of the Stress Process Model.

**Methods:**

The study employed a descriptive phenomenological design Caregivers of children with DD attending the Neurodevelopmental Clinic of a Teaching Hospital were purposively sampled. Data collection involved semi-structured interviews, reaching saturation with 14 participants. The interviews were audio-recorded transcribed verbatim and analysed using thematic analysis.

**Results:**

Four main themes emerged: perception of caregiving, stressors faced by caregivers, negative health outcomes and coping strategies. Perception of caregiving had two sub-themes as stressful nature of caregiving and time-consuming. Six sub-themes were linked to stressors faced by caregivers: the child’s ADL needs, communication barrier, managing challenging behaviour, child’s health needs, unmet educational needs, and economic burden. Negative health outcomes had three sub-themes: decline in physical, mental and social well-being. While some caregivers used maladaptive coping strategies like blaming, others employed adaptive coping strategies like religious coping through prayer, self-encouragement and support from other family members.

**Conclusion:**

The study highlights the complex interaction between caregivers’ perception of their caregiving situation, the stressors they experience, their coping resources,  and the negative health outcomes associated with caregiving. These findings underscore the need for context-specific caregiver programmes to mitigate the negative impacts of caregiving.

**Supplementary Information:**

The online version contains supplementary material available at 10.1186/s12912-024-02142-1.

## Introduction

Developmental disabilities [DD] are long term conditions that significantly impair many domains of a child’s development [1, 2]. Consequently, children with DD face challenges in performing Activities of Daily Living [ADL] such feeding, bathing, communicating, and mobilising [3]. They also experience sleep problems and exhibit challenging behaviours, including aggression, hyperactivity, impulsivity, self-injurious behaviour, and destructive behaviour [4, 5]. These conditions result in multiple long-term impairments, activity limitations, and complex health needs. Unlike other typically developing children, those with DD may require full-time support from caregivers for effectively functioning.

Information on DD is limited in developing countries, but existing evidence indicates a high prevalence, especially in low- and middle-income [LMIC] countries like Ghana [6]. According to Olusanya et al., the number of children living with disabilities in Ghana increased from 241,529 in 1990 to 373,912 in 2016 representing a 54.8% increase [7].

The increasing prevalence of children with disabilities in Ghana implies that many people will become formal and informal caregivers. Formal caregivers, such as teachers and nurses, typically receive payment, formal training, and possess the skills to manage caregiving responsibilities effectively [8]. In contrast, untrained caregivers (informal caregivers) in Africa are often family members like parents, grandparents, siblings, or other relatives [8], are untrained, unprepared, unskilled and unpaid [9]. This is confirmed by studies reporting that caregivers felt they did not have sufficient skills and knowledge to provide the needed care for their child with a disability [10, 11]. They also did not have adequate information about the needs of their care recipients, including rehabilitation [12]. Thus, caregiving puts high demands on these caregivers.

While some studies report that caregivers have positive experiences caring for a child with DD [13, 14] and cope effectively with caregiving demands, others have not. The experiences and outcomes of caregiving in low- and middle-income countries have been largely influenced by factors such as poverty [15] and stigma [16]. For example, the concept of stigma may be more prevalent in settings like Ghana, where people believe that children with disabilities are cursed, bewitched, a punishment from God or other supreme beings, or that their parents have used them for money rituals [17, 18]. Negota and Mashegoane affirm that the biomedical explanation of disability is not considered from caregivers’ perspectives, with the focus instead on spiritual explanations, which deepens the stigma they experience [19].

Other challenges include difficulty accessing social assistance grants, health care and social services, educational and recreational facilities, and infrastructure [12]. For example, some caregivers believe they lack adequate professional support to care for their children with disabilities [12]. Additionally, other caregivers have outlined lack of equipment and assistive devices [12]. Thesee challenges can significantly influence caregiving experiences and lead to increased stress and burden of care. A plethora of studies have documented that caregiving is associated with a high level of stress [20–23]. Stress is pervasive and can influence multiple areas of caregivers’ lives.

The Stress Process Model [SPM] provides a framework for examining the relationship between stress factors and how they interconnect to influence a caregivers’ well-being. The SPM comprises four main components: background and context of stress, stressors, mediators, and outcomes of stress [24, 25]. Background and context are antecedents that influence stress outcomes [25, 26]. Various background factors including caregiver age [27]; caregiver gender [28, 29]; caregiver educational level [30]; marital status [28]; income level [31]; child’s age [27]; child’s gender [32]; caregiving history [33] have been documented to influence the outcomes of caregiving though the evidence is inconclusive.

Stressors are those things that create mental pressure for caregivers, including their experiences, conditions or activities [34]. Primary stressors arise from the care recipients’ needs as well as the care required to address those needs [25]. It also includes behaviour problems, ADL dependencies, burden or overload experienced by the caregiver, functional and cognitive status of the care recipient. These primary stressors may lead to secondary stressors [25], such as strain on the caregiver’s job, social relations, recreational activities and finances.

In the SPM, caregiving outcomes mark the end of the stress process and these are considered the injurious impacts of stressors [24]. Studies have shown that caregivers experience poorer physical health than non-caregivers [35–37], facing issues such as sleep problems [38], low back pain [39], fatigue [40], headache [41], and musculoskeletal pains have been documented. Psychologically, anxiety and depression are commonly reported [31, 33, 42]. Resources like coping mechanisms and social support within the SPM serve as mitigating factors against the negative impacts of caregiving.

While some studies have explored the experiences of caregivers of children with disabilities in the Ghanaian context, they have utilised any theoretical framework and have typically focused on one form of DD [17, 18]. Applying theory in qualitative research can provide a structured framework to guide the study [43, 44], organize data, and elucidate connections between different factors [43]. Primarily, understanding the complexity involved in how the caregiving factors are related and how different stressors lead to negative outcomes is crucial in addressing the challenges faced by the caregivers. Thus, the use of the SPM in this study aims to clearly spell out the factors involved in the caregivers’ experiences and how these factors interconnect and lead to health outcomes. Additionally,  the SPM outlined which factors require interventions to improve caregiving outcomes.

Although the relationship between stress and stress outcomes is well-known, further insight into this relationship can guide future studies and the development of interventions to promote well-being of caregivers. Previous studies have focused exclusively on mothers [19, 45] or fathers of children with disabilities [46]. Despite the fact that most caregiving responsibilities typically fall on mothers, it is also important to highlight the experiences of fathers. Furthermore, to see if the experiences will be different among the various categories of caregivers. To address these gaps, this study represents the first application of SPM to explore the experiences of caregivers using a descriptive phenomenological research design in Ghana. It broadens the scope by including various categories of caregivers and types of DD. Therefore, the aim of the present study was to explore the experiences and coping strategies of caregivers within the context of the SPM.

## Methods

### Design

The study employed a descriptive phenomenological design to explore the lived experiences of caregivers of children with DD. This design was chosen to provide a detailed and systematic description caregivers’ experiences without imposing interpretations.

### Research setting

The study was conducted at the Neurodevelopmental Clinic of a tertiary hospital in Ghana. The Neurodevelopmental clinic is located at the Child Health Department and operates on Mondays, excluding holidays. The setting is the largest tertiary health facility in Ghana, which provides specialised care to children with all forms of DD. As such, the facility was seen as having a high patient load and being accessible for most caregivers, which made it convenient for the current study.

### Participants

Participants for this study were purposively sampled from the Neurodevelopmental Clinic of a tertiary hospital. According to [47], the main premise of purposive sampling is being able to select information-rich cases, which would allow gain in-depth insights into the study. The inclusion criteria were: (1) being a primary caregiver and a parent of the child with DD (2) caregiver who is 18 years and above; (3) having provided care for 6 months or more for the child with DD; (4) having a child with DD who is between 5 and 14 years of age and has received a formal diagnosis of DD; (5) being a primary caregiver who is capable and willing to give consent; and (6) being able to speak either English or Twi language. The exclusion criteria were: (1) caregivers receiving a form of psychological or behavioural treatment or on antihypertensive medication; (2) those facing other major life stressors at the time of the study, such as loss of a spouse or significant other.

### Data collection

The first author with the assistance of the clinic nurse- in-charge, examined the clinic database to identify primary caregivers meeting the inclusion criteria, and contacted them via mobile phone. These participants were provided information about the study, including its purpose, risks, benefits, confidentiality, and anonymity, among others. They were informed that the interview would be recorded, and were given the opportunity to ask questions. Interviews were scheduled during their next clinic visit, where participants were supported to sign the consent form before the commencement of the interviews. The individual interviews were conducted in one of the clinic’s consulting rooms, lasted an average of 40 min and were recorded with a voice recorder. The choice of the setting was thoroughly discussed with the participants and they preferred the clinic setting. Data collection was done using semi-structured interviews, guided by an interview guide developed by the authors (see supplementary file 1). All interviews were conducted between October 2021 to January, 2022.

Sample size in qualitative studies is often not determined a priori [48, 49], however, saturation is usually used. Saturation is described as a point at which information collected becomes redundant and additional data does not significantly impact the study [48]. Saturation was achieved with 14 participants. Nine interviews were conducted in the Twi Language while five were in the English Language.

Data was collected during the COVID-19 pandemic era, however, at the time of data collection the infection rate in the country had significantly decreased. Additionally, no participant during the interview made mention of the any negative impact the COVID-19 pandemic had had on their caregiving role. This suggests that the COVID-19 pandemic did not significantly affect the findings of the current study.

### Data analysis

Thematic analysis was systematically followed to analyse the qualitative data. This method is used to identify, analyse, organise, describe and report themes within dataset [50]. Though a lot of patterns could be identified across any dataset, the focus was on those patterns that were relevant especially in answering particular research questions. The analysis was guided by Braun and Clarke’s six-phase process of thematic analysis which are familiarisation with the data, generating initial codes, generating themes, reviewing the theme, defining and naming the theme and write-up [50, 51].

The essence of familiarisation was to become intimately familiar with the data and to identify information that may be relevant to the research question [50, 51]. The audio-recordings were actively listened to before the transcription began. The first author, who is proficient in the native language (Twi) and English, translated the responses from Twi into English. A professional translator translated the Twi transcripts into English, and another independent translator read the English transcripts, translated them back into Twi and compared them to the original Twi transcripts to ensure accuracy. Manual transcription helped to facilitate deep immersion into the data, noting; breaks, pauses and tones [50]. The transcribed data or transcripts were read severally. While doing this, initial trends, patterns, interesting points and meanings were taken note of.

The next stage involved generating initial codes. Codes are considered as the building blocks of themes. Coding was done to produce succinct, short hand descriptive or interpretive labels for information that may be important to the research question. Codes were brief but offered sufficient details to stand alone [50]. In the next stage of data analysis, themes were generated based on the codes. This phase involved reviewing and analysing different codes, and possible combination of the codes to form an aggregated meaning and subsequently a sub-theme or theme. A thematic map was generated. Review of the initial themes was done in relation to the coded data items and the entire data set [51]. During the review of the themes, themes found to be overlapping were collapsed. The themes were then defined and named. The names were concise, informative and memorable. During the write-up stage, themes were built in a coherent manner. They were organised such that they built on previous reported themes.

### Ethical considerations

The study obtained approval from the Human Research Ethics Committee (HREC) of the University of Witwatersrand. The Scientific and Technical Committee also approved it, as did the Institutional Review Board of the Korle-Bu Teaching Hospital, with approval numbers KBTH-STC/00021/2021 and KBTH-IRB/00021/2021 respectively. The study adhered strictly to principles of informed consent, privacy, confidentiality, respect, the right to withdraw and responsibility. Minor participants were not included in the current study, the focus was on adult participants.

### Trustworthiness of the study

For a study to be considered trustworthy, clear procedural rigor is essential [52]. The criteria commonly used are credibility, dependability, confirmability, transferability, authenticity and reflexibility [53]. To ensure credibility, this study employed established qualitative research methods, careful comparison with similar projects, engaged in prolonged interactions between the first author and participants, and obtained consent from all participants [52]. Iterative questioning was used to uncover deliberate lies, and debriefing sessions were conducted between first author and the other authors. Member checks were conducted at every stage of data collection to ensure accuracy and avoiding falsehoods. Transcripts of dialogues between six participants and the first author were provided to verify their intentions. Dependability, which refers to the stability of data over time and study conditions [54], was ensured through the use of inquiry audit technique [52]. To establish confirmability, the detailed recording of the research interview transcripts; raw data field notes including the date and time of the interview, how consent was obtained, and the process of the interviews were described. Transferability was achieved by giving a detailed description of the research design, methods, and processes applied [54]. Reflexibility was maintained by the authors keeping a diary to examine how their own assumptions, beliefs, and values could influence the research decisions.

## Results

### Sociodemographic characteristics of caregivers and care recipients

The sociodemographic characteristics of the caregivers and children with DD are described in terms of caregiver age, gender, marital status, occupation, duration of caregiving and relationship to the patient. The findings from Table 1 indicate that both caregivers and children with DD in the study were relatively young. The majority of the caregivers were females (*n* = 11), cohabiting or divorced or unmarried (*n* = 9), unemployed (*n* = 8). Additionally, majority of caregivers had provided care more than 2 years (*n* = 11). Most children with DD were males (*n* = 9), with CP (*n* = 4) or ASD (*n* = 4) being the most common diagnoses. Detailed information is presented in Tables 1 and 2:

**Table 1:** Socio-demographic characteristics of caregivers and children with developmental disabilities

Participant	Gender	Age	Marital status	Employment status	Duration of caregiving	Gender of child	Specific diagnosis
1	Female	31	Married	Employed	8 months	Male	CP
2	Female	35	Single	Employed	2 years	Male	ASD
3	Male	42	Married	Unemployed	2.5 years	Female	DS
4	Female	27	Cohabiting	Unemployed	5 years	Female	ADHD
5	Female	24	Single	Employed	3 years	Female	CP
6	Female	28	Married	Unemployed	2 years	Male	ASD
7	Male	36	Divorced	Employed	3 years	Male	DS
8	Female	34	Single	Employed	4 years	Male	ID
9	Female	35	Married	Unemployed	2.5 years	Female	CP
10	Female	29	Married	Unemployed	7 years	Male	ADHD
11	Female	30	Cohabiting	Unemployed	3 years	Male	ASD
12	Male	39	Single	Unemployed	2 years	Male	ID
13	Female	44	Divorced	Unemployed	8 years	Male	ASD
14	Female	26	Married	Employed	1.8 years	Female	CP

**Table 2:** Mean age of caregivers and children with developmental disabilities

Caregiver’s age	Range (years)	
	Mean (years)	32.85
	SD	6.48
**Child’s age**	Range	
	Mean	6.86
	SD	2.03

### Themes, sub-themes and categories emerging from the study

The description of themes, sub-themes and codes in the study is summarised in Table 3.

**Table 3:** Themes, sub-themes, and codes

Themes	Subthemes	Codes
Perception of caregiving role	Stressful nature of caregiving	Overwhelmed with caregiving Tiredness from caregiving role Difficulty combining caregiving role and other social roles
	Time consuming	Caregiving takes a lot of time Caregiving is extended Caregiving takes away one’s leisure Caring for a child with DD is a task that needs time
Stressors	Child’s ADL needs	Assisting with feeding Assisting with bathing Assisting with elimination
	Communication barrier	Caregiver communicating expectations Child with DD communicating his or her needs
	Managing challenging behaviour	Forms of difficult behaviours Triggers of difficult behaviours Dealing with usual behaviours
	Child’s health needs	Proximity of the hospital to caregiver’s home Visiting the hospital many times Preparing for hospital appointments Difficulty obtaining prescribed medication from hospital pharmacy Time spent at the hospital during visits or appointments
	Unmet educational needs	Difficulty getting a special school for the child with DD Proximity of special schools High cost of enrolling in special school Gaining admission into mainstream school Attitude of teachers from mainstream school
	Economic burden	Cost of medications Cost of assistive devices Cost of other therapies Loss of job Conflicting demands of job and caregiving Under employment Decreased output at work
Negative health outcomes	Decline in physical well-being	Low back pains General body pains Headaches Fatigue Difficulty in sleeping at night Interrupted sleep at night
	Decline in mental well-being	Decreased concentration Feeling angry Feeling sad Feeling anxious
	Decline in social well-being	Unable to take part in social functions Limited social contact Loss of interest in social activities Negative attitudes from other people Strained relationship with partners Strained relationship with friends Strained relationship with community members
Coping strategies	Adaptive coping strategies	Religious coping through prayer Self-encouragement Support from immediate family/nuclear family
	Maladaptive coping strategies	Blaming self Blaming others Blaming supreme being

**Fig. 1:**
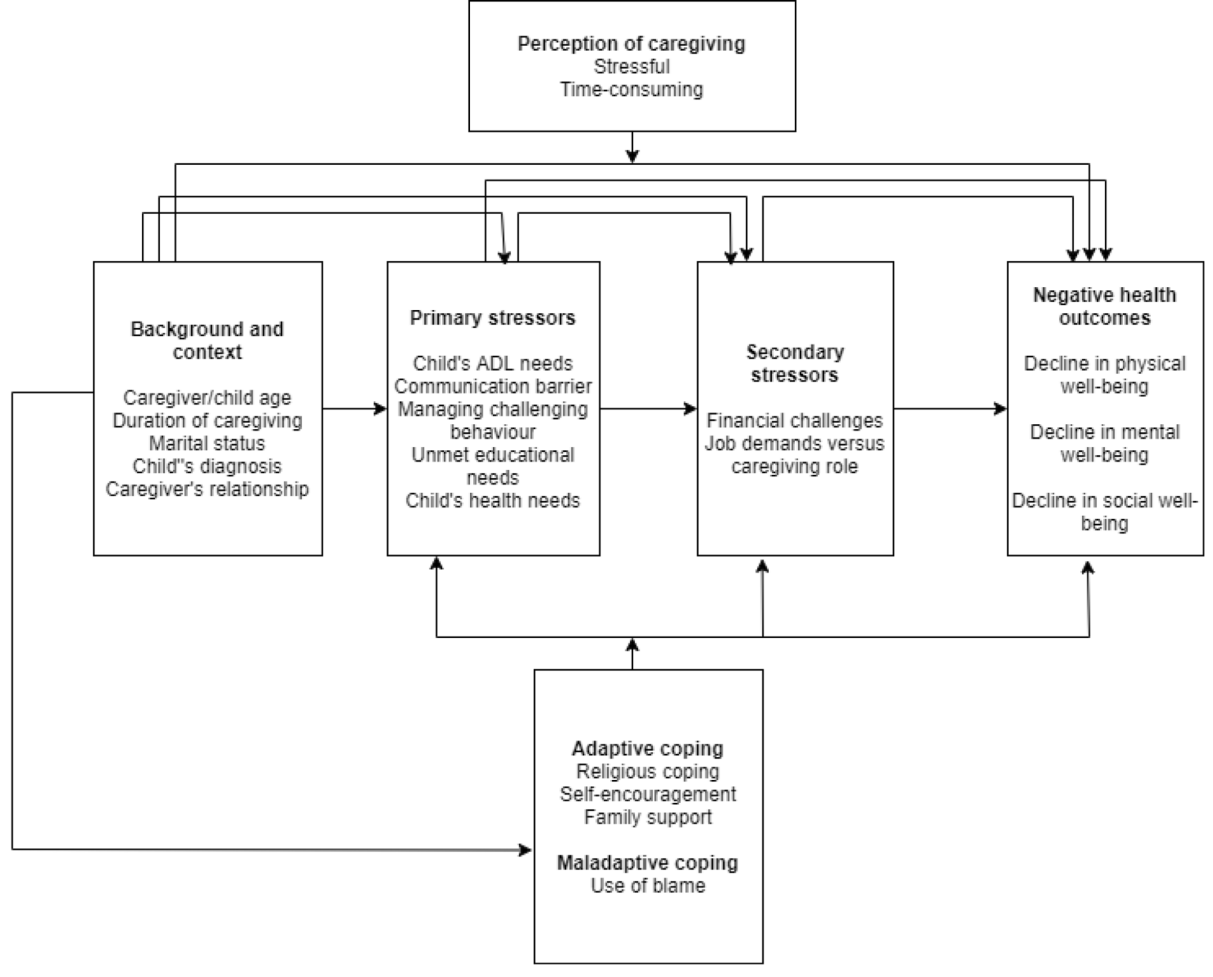
Findings of phenomenological study examined in context of SPM

The findings of the study were examined in the context of the Stress Process Model, which served as the theoretical underpinning. It was observed that caregivers experienced both primary and secondary stressors, consistent with as the SPM. Furthermore, poor health outcomes were also reported, similar to the SPM. Caregivers used certain resources: spirituality/prayer, support from family and self-encouragement to cope with the stressors of caregiving. How the current study fits into the SPM is outlined in Fig. 1:

## Theme 1: perception of caregiving role

Generally, caregivers perceived that caring for a child with DD as negative. Two sub-themes were generated: caregivers perceived caregiving as stressful and time consuming.

### Sub-theme: stressful nature of caregiving

Caregivers were overwhelmed with the caregiving activities for children with DD, leading to high levels of stress, fatigue and exhaustion. The level of dependence of children with DD on the caregiver comparable to that of a typically developing children did not appear to be the same. Children with DD exhibited greater dependency on their caregivers, had extra needs and required adequate support to thrive. A caregiver had this to say:

*“Caring is stressful. I have other children, so I usually compare them, and you notice that as the “normal” is growing they begin to acquire some level of independence and are able to do some little things for themselves. In the case of the child with the disability, it’s not like that. You do everything for them.” (P5, mother, 24 years)*.

Performing activities of daily living (ADL) for the children with DD was particularly stressful for caregivers especially in the absence of mechanic devices. These activities are performed daily and it would be impossible for the caregiver to skip or ignore. Any attempt by the caregiver not to perform such activities imply, the child with DD will suffer. The inability to accomplish essential activities of daily living may lead to unsafe conditions and poor quality of life. A female caregiver with a child with CP recalled:

*“The experience in taking care of the child is difficult. As you know, as a caregiver, I do everything for the child. If I don’t, meaning the child won’t feed, bath, dress and so forth.” (P11, mother, 30 years)*.

Some other caregivers shared that performing multiple roles in addition to being a caregiver was stressful. The caregiver had to work either full time or part time in order get some money to take care of their child with special needs. Social roles like taking care of other children and their partners made caregiving quite stressful. One caregiver narrated:

*“This task is not easy at all. Taking care of such a child is full time job. But you cannot also say you won’t work. Where will the money be coming from to take care of the child? You have to work; you have to take care of his siblings and you have to perform your role as a wife. In one instance I am a caregiver, in another a mother and in another a wife. All these come with responsibilities.” (P14, mother, 26 years)*.

### Sub-theme: time-consuming

Other caregivers were concerned with the time-consuming nature of caregiving. The activities performed during caregiving took caregivers time. In the opinion of some caregivers, the activities start in the morning and continues till the evening. One caregiver remarked:

*“I spend a lot of time in taking care of this child. Once you start the day’s activities, by the time you realise it’s evening.” (P3, father, 42 years)*.

Caregivers barely had time to take care of themselves. Many of them channelled their energy and time in taking care of their children with DD. Possibly as result of children with DD not being able care for themselves and caregivers had to do that for them. One caregiver narrated her experience in the following:

*“Caring for my child takes all my time. Everything is almost about him now. To be frank, I hardly get time for myself these days.” (P10, mother, 29 years*.

## Theme 2: stressors faced by caregivers

Caregivers had stressors that increased their burden of care and subsequently affected their well-being. The sub-themes generated were child’s ADL needs, communication barrier, managing challenging behaviours, child’s health needs, unmet educational needs, and economic burden.

### Sub-theme: child’s ADL needs

For this sub-theme, caregivers felt responsible for making sure that their children were comfortable by ensuring they provided them with all their needs, however, this was a major stressor to caregivers. Caregivers had to handle tasks like feeding, bathing and assisting with elimination because their children could manage these activities independently. This is explained in detail below:

#### Feeding

Caregivers encountered challenges when feeding children with DD. For some caregivers, children with DD preferred certain types and textures of food. Feeding sessions also took longer than usual, and any attempt to rush through meals resulted in negative consequences such as choking. Despite trying various types of food, caregivers found that their children often struggled to tolerate new options, making them hesitant to introduce unfamiliar or new foods. A caregiver had this to say:

*“My daughter can only take liquid diets. If you’re not careful and you overfeed or don’t feed well, she will vomit everything. Because of this, I am always very careful when feeding, so it takes a while to finish with one feeding. If you do not feed her well too, she will be crying throughout the day because she is hungry. Anytime I try solid food, it is as if she is choking, so I have stopped.” (P5, mother, 28 years)*.

Children with DD often had specific food preferences and would refuse any food outside their preferences. Thus, in some instances they could not be fed with what the entire family would eat. This meant extra work and cost to the caregivers as they had to spend extra money to prepare different types of foods tailored to their child’s preferences. One caregiver narrated her experience in the following:

*My child prefers only smooth foods. If the texture of the food is not what she wants, she will reject it. No matter what you do, he will not eat it. So most often, I have to prepare his food differently from what the whole family is coming to eat.”(P13, mother, 44 years)*.

Some caregivers had successfully trained their children to perform some activities of daily living, however, those activities could not be performed without constant supervision. Regarding feeding, some children could feed themselves but spent more time in doing so. Apart from the time spent, the children with DD spread food particles all over, creating additional work for the caregivers after every feeding episode. A caregiver remarked:

*“In terms of his feeding, it is not easy. He will soil himself with the food, a large quantity of food particles will be spread on the floor after eating. He will not also allow me to feed, but we end up taking a lot of time because I have to supervise. After feeding, what spreads on the floor is greater than what goes inside.” (P11, mother, 30 years)*.

#### Elimination

Caregivers mentioned they had difficulty regulating elimination needs for children with DD, as these children could not communicate when they needed to urinate or defecate. Sometimes, immediately, after bathing them, they needed to be cleaned again because they have soiled themselves. One caregiver illustrated:

*“My son is not 100% strong compared with the elder sibling, who is not facing any challenge. Due to his condition, taking care of him is very difficult. One problem is his inability to inform me when he wants to urinate or defecate. He totally depends on me. Sometimes after bathing, he soils himself.” (P1, mother, 31 years)*.

The use diapers had become part of caregivers’ strategies to deal with issues of elimination among children with DD. Its usage was more preferable as non-use was associated with more work and stress for the caregiver. One caregiver narrated:

*“He is always in diapers because you cannot be sure when he will want to urinate or defecate. If you allow him to soil himself, usually that is more difficult work, compared with him having diapers on.” (P12, father, 39 years)*.

#### Bathing

Bathing was another activity of daily living caregivers found challenging . For those children with DD who have movement and coordination issues, the caregivers had to physically carry them to the bathroom. The issue of gaining the cooperation of children whilst performing activities of daily living is a concern for caregivers. For them, without cooperation, more time is spent on performing certain activities including bathing. A caregiver’s experience is excerpted in the following:

*“He cannot walk. So, you have to carry him to the bathroom. A lot of time is spent because sometimes there is no cooperation and for a shared bathroom, I worry a lot.” (P2, mother, 35 years)*.

Some caregivers bathing their children with DD in basins so as not to cause undue stress for the child with DD. A caregiver recalls her experience as:

*“I most often bathe her in a basin and afterward go and pour the water away. When I take him to the bathroom, she cannot stand, and I cannot let him sit on the bare floor, so that is the strategy I use. As she is maturing, I am becoming more concerned about this.” (P9, mother, 35 years)*.

### Sub-theme: communication barrier

Caregivers struggled in communicating their expectations to the children with DD. There are things they expected the children to do but they did not seem to understand the expectations of the caregivers. Caregivers had resorted to inappropriate ways of communicating to the children with DD such as screaming or shouting. A caregiver shared her experience as follows:

*“Caring for him is difficult because, currently, he is not communicating, so you have to be very smart and be able to read his patterns. When I want him to do something, it’s difficult to tell him. Sometimes, regardless of how hard I try, he just seems not to get what I am saying. I end up screaming. Screaming has now become part of the way I talk to him. I know it is wrong, but I don’t know how to get him to either do something or stop doing something bad or negative.” (P8, mother, 34 years)*.

Other caregivers observed that the children with DD had difficulty communicating their needs to them, leading to misunderstandings and feelings of helplessness among caregivers. Two caregivers gave the following account:

*“I am trying hard to learn how he communicates his needs, but it looks like I have not gotten there yet. I sometimes feel so helpless when I am unable to figure out what exactly he needs or wants. Sometimes it’s like he’s demanding something and I also do something else.” (P12, father, 39 years)*.

*“This child does not talk, nor is he able to express his mood or feelings to you for you to be able to identify the problem he is facing and the necessary help you need to offer.” (P1, mother, 31 years)*.

### Sub-theme: managing challenging behaviours

Caregivers reported that inappropriate behaviours in children with DD were a significant source of stress. When these children are idle, they often exhibited challenging behaviours like hyperactivity. Findings ways to keep the children meaningfully engaged was essential to reducing such challenging behaviours. A female caregiver’s experience is excerpted in the following:

*“I have experienced a lot of challenges. The child easily messes his immediate environment; he fights with people around him; and he becomes hyperactive. He makes you complain and correct him over and over again because of his hyperactivity. He usually thinks his actions are right, but they end up damaging things around him. He is distracted when he watches cartoons on the television set, before I can also concentrate on my house chores.” (P10, mother, 29 years)*.

Caregivers reported some level aggressiveness in their children with DD. Challenging behaviours were triggered by some factors. Identifying the triggering factors and dealing with them was a good way of manging children with DD. Thus, it is the responsibility of caregivers to identify and manage triggering factors which will help decrease the incidence of challenging behaviours. One caregiver, for instance, indicated that hunger in her child with DD triggered aggressiveness. She gave the following account:

*“She sometimes becomes aggressive. I observed that this happens when she is very hungry. So, I ensure there is always food available, and I don’t let her get hungry. I have timed her feeding pattern so I know the right time to feed him. However, there are times she will stop eating and may not really be satisfied; that is when she will display aggression, though it may not be time to feed her.” (P9, mother, 35 years)*.

Caregivers observed that children who exhibit challenging behaviours need constant monitoring and supervision in order to prevent them from injuring themselves or others. A caregiver recounted:

*“My child is very hyperactive and it is almost impossible to let him sit for a few seconds. He is always up, doing something. He is here, and the next moment he is there. You have to be monitoring him continuously; otherwise, he may injure himself before you can even tell. I keep chasing him around and screaming. That alone takes a lot of energy. It really drains me and makes me exhausted”. (P11, mother, 30 years)*

### Sub-theme: child’s health needs

Caregivers understood the importance of seeking medical attention for their children with DD and considered as one of the priority areas for them. However, preparing for hospital appointments required significant planning and effort. Hospital visits implied caregiver could not engage in any other activity. This is because they spent most of the time at the hospital and returned home late. A caregiver had this to say:

*“When you have hospital appointments, you cannot say you will miss them because you think about your child first. You have to wake up very early and prepare. When you get to the hospital, you may end up spending almost the whole day there.” (P4, mother, 27 years)*.

Proximity of the hospital was a problem mentioned by caregivers. Some caregivers had to travel from other regions to the capital city in order to access health care for their children with DD. Getting prescribed medications created a challenge for them as the medications were sometimes unavailable at hospital pharmacy. A caregiver recounted:

*“Anytime we have an appointment, I begin to think, and it makes me anxious. Travelling to the hospital, going through the OPD, meeting the doctor, going to the laboratory, going for the medications, and travelling back home is stressful. Sometimes getting the medications is really a struggle, you may not get some at the hospital pharmacy, so you have to roam from pharmacy to pharmacy just to get what has been prescribed.” (P11, mother, 30 years)*.

### Sub-theme: unmet educational needs

Caregivers struggled to access quality education and maintain their children with DD in school. They would have preferred if their children could easily have some form of special education, which they believed would have assisted the child in developing certain key skills. Unfortunately, it does not appear so, and hence, caregivers identified the unmet educational needs of their children with DD as a stressor. They were concerned about enrolling the children in school and the challenges associated with keeping them there.

Caregivers expressed worries about finding the appropriate schools for their children, for them inclusive education would have been ideal. However, the concept appears not to be practical and most mainstream schools rejected children with special needs. Therefore, caregivers were usually forced to consider special schools for their children with DD. Unfortunately, special schools were few, expensive and inaccessible to a lot of caregivers. Some caregivers expressed their concerns as follows:

*“I really had a tough time enrolling her in the mainstream school. In Ghana, we always talk about inclusive education, but I doubt if it is really working. No school wants to accept him. It makes me consider special schools, but these schools are not common, especially the government-based ones, and the private-based ones are also quite expensive.” (P3, father, 42 years)*.

*“Special schools are expensive. The one I know of is not so close. You have to pick up about three vehicles before you get there. In government schools, once they know the child has a problem, they won’t even admit him or her. But I believe when he is enrolled, at least he will be taught some basic things that will be useful at home.” (P8, mother, 34 years)*.

Due to the unique nature in addressing the needs of children with DD, some teachers in mainstream schools often complained about the behaviour of children with DD. Caregivers were not comfortable receiving constant negative reports about their children. One caregiver illustrated:

*“There are too many complaints that sometimes I can’t bear. Today your child did this; tomorrow your child has done that. Every day and its story. This can be really stressful. So, once he goes to school, I keep wondering what complaints I will receive when I pick him up from school.” (P6, mother, 28 years)*.

Caregivers attributed the constant complaints by teachers in the mainstream schools to the inadequate knowledge they had on managing children with special needs. There are teachers who have been specifically trained to understand and manage children with special needs. Inadequate knowledge may translate into poor skills leading to frustration on the part of the teacher and finally constant complaints. One caregiver had this to say:

*“Teachers complain a lot about my child’s behaviour in school, but I also think it’s because the teachers do not have much knowledge in taking care of such children. You can’t blame them. Taking care of one is difficult, so in a class where you have 30 to 40 children, it’s going to be extremely difficult. And you cannot also spend time on only one child.” (P11, mother, 30 years)*.

### Sub-theme: economic burden

Caregivers explained that caregiving had a negative impact on their finances as they invested money in the pre-diagnosis, diagnostic, and post-diagnostic stages. All caregivers were at the post-diagnostic stage, where their children had received a definite diagnosis from the specialist. The caregivers were overwhelmed with the cost of medications, other therapies and assistive devices.

Caregivers did not receive any form of financial support and they recounted the medications were expensive. The National Health Insurance Scheme did not cover all the medications, usually brands of medications covered were not preferred by the caregivers. They shared their experiences as follows:

*“The medication for the child is expensive and is really draining me financially, especially considering the fact that I have no form of support and my income is also nothing to write home about. When they get finished and you do not have money, it is a big problem. The medications I buy are very expensive. Sometimes the National Health Insurance covers some of the medications, but those are not the original medication brands prescribed by the doctors. I usually do not go for those ones. I usually buy the original medication brands. Monthly, I spent not less than five hundred Ghana Cedis (GH¢ 500.00) on medication. The greatest challenge is money to buy those medications to last for a month.” (P13, mother, 44 years)*.

Apart from medications, other services like physiotherapy was expensive. A caregiver had this to say:

*“There is physiotherapy, there are other devices that we buy as well as medications. These are very expensive. Sometimes, there is no money to cater for her and her other siblings.” (P9, mother, 35 years)*.

Some concerns were raised about lifting the children, especially in the absence of assistive devices, which could have made it quite easy for the caregivers. For caregivers with little or no financial support, purchasing assistive device may be difficult. The challenge involved with lifting increases as the child with DD ages which is associated with weight gain. Increased weight requires caregivers to exert a lot of energy to lift their children with DD. A caregiver had this to say:

*“I have not been able to purchase a wheel chair for easy movement around, so I end up carrying him. Initially, it wasn’t so stressful. But now, day in and day out, he is gaining weight, so it is becoming increasingly difficult to carry him and be lifting him around like I used to do.” (P1, mother, 31 years)*.

The job of caregivers was directly linked with their income levels. Being underemployed or finding difficulty balancing work and caregiving responsibilities often had a negative impact on their finances. Fully employed caregivers frequently had to request time off from work for hospital appointments. This affected their efficiency at the work place, however, they had no options. It was also likely to lead to their dismissal especially if the institution is private. One caregiver asserted:

*“I am a nurse, and I am supposed to be on duty. I frequently ask permission to absent myself from work. My employer knows my child’s condition, so I am able to seek permission so I can attend to her or bring her to the hospital. My employer does not complain whenever I ask permission, but I know others will complain.” (P3, father, 42 years)*.

Self-employed caregivers had difficulty balancing their caregiving role with their job. They believed their role had caregivers interfered with their jobs. Caregivers had to desert their jobs which was the only option. This could afford the caregivers ample time to perform their caregiving roles. The narratives below throw more light on these reports:

*“I used to be a seamstress, but now I have stopped because of him. He takes all my time. I don’t want to be in a situation where I take people’s clothing and I won’t be able to sew for them. Besides, taking care of him is quite stressful.” (P6, mother, 28 years)*.

*“Caring for him has affected my job. I was a local contractor who was hustling to take care of the family. Now I don’t get the chance to move around like I used to do before.” (P12, father, 39 years)*.

*“She is unable to stand or sit, so even if you get a work, you can’t leave her and go. Currently, I am not working. I use to sell but no more.” (P5, mother, 24 years)*.

## Theme 3: negative health outcomes

Caregivers mentioned that their caregiving role affected them negatively. Three sub-themes were generated: decline in physical, mental and social well-being. In terms of the decline in physical well-being, caregivers mentioned pain-related effects, fatigue, and compromised sleep. The decline in mental well-being was marked by lack of concentration, feelings of anger, sadness and anxiety. Sense of social isolation and changes in social relationships were highlighted in the sub-themes of decline in social well-being.

### Sub-theme: decline in physical well-being

The participants highlighted that caregiving had multifaceted impact on their physical well-being. Caregivers’ ability to perform physical activities and carry out social roles were hindered by physical limitations and experiences of low back pains, body pains, fatigue, poor and compromised sleep. Caregivers observed deterioration in physical well-being, which hampered their ability to achieve optimal functioning. Thus, carrying out normal daily tasks was marked with exhaustion and discomfort.

#### Pain-related effects

Pain-related effects were mentioned by caregivers. They mentioned they experienced low back pains and body pains which they attributed mainly to carrying the children with DD around whilst performing other activities. Two caregivers shared their experiences as:

*“You know…. you have to carry her wherever you go. When I am going to church, she’s at my back. When I am going to the market, she is at my back. When I am going to the hospital, she’s at my back. She cannot walk so you have to carry her. I have low back pains and feel very tired. The only instances I do not carry her is when there is someone at home especially the father. Even in those instances, I am in a hurry to come home.” (P5, mother, 24 years)*.

*“It is something I did not expect but it has come. Sometimes I really find it difficult. Caring for him has been stressful. I experience body pains, backpains.” (P9, mother, 35 years)*.

Caregivers experienced body pains and resorted to taking over-the-medications. Though the caregivers knew taking unprescribed medications had negative consequences, they had to take the medications. Other caregivers found some time to rest. The essence of taking the medications and resting was to ensure the caregivers had renewed strength for the next day’s caregiving activities. Two caregivers shared their experiences as:

*“Sometimes, I feel very tired with a lot of body pains. You have to be lifting, feeding, bathing and do almost everything for him. You know I have two other children, so combining all those tasks makes it difficult. I end up relying on pain killers just to make sure I have enough strength to carry on for the next day. I know too much of pain killers is not good especially when they are not prescribed by the doctor but I’m unable to stop.” (P3, father, 42 years)*.

*“She is very heavy, carrying her leave me with a lot of body pains. Sometimes it is like you have been beaten. I carry her throughout the day unless of course she’s sleeping. If not, she’s at my back. She doesn’t like going to other people. If I attempt, she will cry uncontrollably. I sometimes take pain killers, other times I try to sleep when she’s also asleep.” (P9, mother, 35 years)*.

#### Fatigue

Beyond the low back pain and the body pains, the caregiving demands affected the caregivers with some reporting palpitations and exhaustion. They attributed the above to their inability to get adequate rest and sleep for themselves. One caregiver stated below as:

*“Sometimes, I get palpitations, my heart beats very fast. This I am sure is as a result of the stress in taking care of him. You know you don’t actually get adequate time for yourself. The attention shifts from yourself to ensuring that your child has the best. Then you end up getting drained.” (P8, mother, 34 years)*.

Some caregivers had to visit the hospital for medical attention because their experiences were beyond what they could manage at home. For some caregivers they were given medications while others were advised to reduce the stress. To reduce caregiving stress and burden, caregivers have had to rely on spousal support. Two caregivers shared their experiences as:

*“After feeding, bathing, and doing all the other tasks I feel very exhausted. My heart beats so loud. At a point I had to even visit the hospital, to go for medications.” (P11, mother, 30 years)*.

*“I sometimes experience palpitations from my inability to sleep at night and also the stress in caregiving. I have even visited the hospital to check my blood pressure. They advised me to reduce my work load. Sometimes my husband supports but you cannot leave everything for him to do. I end up performing most of the tasks.” (P6, mother, 28 years)*.

#### Compromised sleep

Caregivers observed that there had been changes in their sleep patterns, they did not have adequate sleep and most at times stayed awake during the night. The caregivers did not sleep because of poor sleep patterns in their children with DD. It was extremely difficult to sleep during the day as well as they were engaged with caregiving activities. Two caregivers commented:

*“Sometimes, I become very restless. Sometimes in the night when I am sleeping, I have to get up just watching him, looking at him. Thus, most at times whilst he is sleeping, I am awake. You know that in the day I can’t sleep because of the caregiving roles, so once I am unable to sleep at night, then during the day the work continues. So, the cycle of not having adequate sleep at night is really draining me. Most at times, I feel restless. I need adequate sleep.” (P8, mother, 34 years)*.

*“My son prefers to be active during the night, so you can imagine. How can you comfortably sleep whilst he is awake? It is practically impossible. When he’s awake, you also have to be awake. When he sleeps, you also sleep. Most nights I don’t have a good sleep and during the day I’ll be at work so no time to have some nap during the day.” (P7, father, 36 years)*.

The challenge of some children with DD not being able to sleep well at night and being overly active at night had been reported to the hospital. However, the side effects of the prescribed medications deterred the caregivers from adhering to the right time of medication administration. A caregiver narrated:

*“As for having adequate sleep, it is out of the picture. He does not sleep. He could go a week without sleeping well at night. In instances when he sleeps at night, it is very short. I am thinking, he thinks when he sleeps, I will run away. So, I am unable to sleep and I get tired. I even reported at the clinic about his inability to sleep and they gave him some medications. However, the medications make him very weak so I don’t often give them to him.” (P6, mother, 28 years)*.

### Sub-theme: decline in mental well-being

Caregivers mentioned that there were some changes in their mental well-being. They mentioned they could not concentrate, they experienced sadness, anger and anxiety. Additionally, they blamed themselves and others.

#### Lack of concentration

Caregivers expressed that they could not concentrate and were easily forgetting things because their thoughts were always flooded with that of their children. It caused them to make some unavoidable mistakes on their job. They shared their experiences as:

*“It’s too tough, it has really affected me to be frank psychologically it has affected me. It is something I did not expect but it has come. it’s so frustrating and I can’t concentrate. At work I easily lose focus of what I am doing. Sometimes some mistakes are so avoidable but I still go ahead and make them because of lack of concentration.” (P2, mother, 35 years)*.

*“It’s interesting how I easily forget these days. Sometimes, I’ll keep something in the room. Only the next day I try to recollect where I have kept them, I don’t even remember where exactly I placed them. I have to search and search till I find them or sometimes even forget about the searching because I am unable to find the thing.” (P4, mother, 27 years)*.

*“For me, I am unable to think clearly. My thoughts are always flooded with a lot of questions bothering on my child’s condition. I ask myself if ever my child will walk, will be able to have a fulfilling life. This even becomes worse when I see other “normal” children who are of my child’s age.” (P8, mother, 34 years)*.

#### Anger

Caregivers admitted that expressing anger was a natural response to situations, however, the frequency with which they expressed anger was beyond normal. The issues were not things they needed to be angry with. Some had this to say:

*“I am a well composed person. I used to hardly react to issues. It was difficult to make me angry but off late I noticed I am losing myself. I am no longer me. I can’t find myself any longer. Why do I say so? It’s because I get angry when I truly I am not supposed to be. I guess it is as a result of the stress I am going through.” (P 11, mother, 30 years)*.

*“It’s normal to be angry once a while. But when it becomes continuous, then you can say that something is really wrong. I get angry more often and I know this is not good for my health. I don’t know if I am right but I have heard that getting angry often may give you high BP.” (P7, father, 36 years)*.

Caregivers were mainly angry towards themselves or others. The frequent expressions of anger had affected their relationship with their immediate family. One caregiver illustrated:

*“I easily become angry these days. It used not to be like that. I noticed that with the least provocation then I get angry. I think it’s even affecting those around me in a way. They feel I would scream or shout at them so they as much as they can to avoid having contacts with. Sometimes I would regret my actions but most often you can turn back the hands of time.” (P2, mother, 35 years)*.

#### Sadness

Caregivers felt sad and at times cried. This feeling is usually triggered when caregivers are alone or isolated. Two caregivers illustrated:

*“I don’t know how to describe how I sometimes feel to you. I sometimes feel very sad. I can be in the room crying and asking myself a lot of questions. The crying does not make me feel better but I become sadder.” (P5, mother, 24 years)*.

*“The thoughts of having to deal with this difficult situation really makes me sad. You see when you started asking me questions, I began to have tears in my eyes. When I am outside, I try to show that I am strong but the story is different when I am alone. When I am alone, I feel sad and cry a lot.” (P4, mother, 27 years)*.

The feeling of sadness was attributed to stress they were going through and the stigma they face from society. A caregiver shared her experiences as:

*“I feel down most of the times. Not knowing what the future holds and if really, I will be able to go through all these stresses. The society even makes it worse. The comments they will make are so painful. Some of them when you hear them you just can’t control yourself, the only response that comes is tears flowing down your eyes.” (P13, mother, 44 years)*.

#### Anxiety

Another theme that evolved was caregivers being anxious about how the caregiving process was going to evolve. P2 and P10 expressed their views as:

*“Personally, I don’t know what the future holds for my son. I keep thinking about how our lives, I mean myself and my child, will evolve.” (P2, mother, 35 years)*.

*“When you cannot predict what will happen to your child it’s really disturbing. Sometimes you feel like your hopes and aspirations have been brought to a halt.” (P10, mother, 29 years)*.

### Sub-theme: decline in social well-being

Caregivers expressed that caregiving affected their social interactions and relationship. Caregivers mentioned that they felt isolated and were unable to take part in social functions because it was difficult to manage their children in public and also because of the negative attitudes from the public. Hence, to avoid these, they stay at home. Some caregivers had this to say:

*“I hardly go for social functions like funerals, weddings and others. If the ceremony is not really close, I mean if it is not family-related ceremonies you won’t find me there. I stay at home most often. When you take her, you may have to feed her, she will soil herself and yourself as well. She will also cry especially when she sees crowd and all attention will be drawn towards you. Some people will say take her for walk and all that. To avoid all of these, I stay at home.” (P2, mother, 35 years)*.

*“I have lost interest in the activities I use to enjoy, so most often you would find me indoors. I tell myself it’s better not to hear anything form people which will irritate me and create problems for me.” (P9, mother, 35 years)*.

For some caregivers, they defied all odds and attended public gatherings, however, they kept to themselves during these gatherings. So, though they were in public, they were still socially isolated. A caregiver narrated:

*“I can’t mingle as I used to. I have a feeling when I approach people they talk about my situation and my ridicule me when I am not even around. Because of that I do go out very often. When I do, I try as much as I can to keep to myself so that people do not insult or laugh at me.” (P5, mother, 24 years)*.

Caregivers mentioned that there had been changes in their social relationships. The changes had affected the relationship between their partners/spouse, their families, friends and society at large. Some caregivers narrated their experiences as:

*“As for the rejection and the stigma it is not easy. Initially, there was a battle between myself and my husband’s family. They said they don’t have such children in their family and that it was coming from my family. As it stands now, I don’t have a cordial relationship with them like I use to have. Even now my husband is also behaving in some way, anytime I ask for money to buy something for the child, he tells me he is not having money which I know he has.” (P9, mother, 35 years)*.

*“Once your child is a special child, you lose your friends. Currently speaking, I don’t have friends. All my friends are gone. They just don’t want to have anything to do with you any longer through no fault of yours.” (P6, mother, 28 years)*.

*“There is so much discrimination. Society makes you feel there is something really wrong and as a result of that you and the child should not come close to people. The public stares are enough to bring you down any day.” (P14, mother, 26 years)*.

## Theme 4: coping strategies

Caregivers expressed how they have been dealing with the stress they go through. Some coping strategies were adaptive, others were maladaptive. One adaptive coping mechanism frequently mentioned was prayer. They expressed that anytime they felt overwhelmed, they resorted to prayer, and the prayer really made them feel good afterwards. Others expressed that they encouraged themselves. Some caregivers also had some support from their families. Other caregivers blamed themselves, others, or God as a way to deal with stress.

### Sub-theme: adaptive coping

Caregivers included in the study relied so much on prayer to a higher being. They mentioned it was an avenue to pour their hearts out. They felt better after prayer because they got the opportunity to talk about their problems not with man but God. Some caregivers had this to say:

*“…. What has really helped me all these periods is prayer. I pray a lot by myself. Anytime I pray, I feel relieved as if a burden has been lifted from me.” (P8, mother, 34 years)*.

*“One thing that has really helped me cope is prayer. In instances where the feeling is uncontrollable but, at the same time, you have no one to talk to, the option becomes God. And for God, the only way we can talk to him is through prayer.” (P9, mother, 35 years)*.

Encouraging themselves as caregivers was also used as a coping strategy. The caregivers used the opportunity to talk to themselves about the situation. The encouragement was mainly based on positive things. Some caregivers expressed their views as:

*“I encourage myself a lot. I tell myself; it shall be well with me and my child.” (P10, mother, 29 years)*.

*“If you don’t encourage yourself, nobody will do that for you. I talk to myself a lot and encourage myself. After all, I can’t change the situation.” (P12, father, 39 years)*.

Caregivers were able to cope due to the support of their partners and children. Some partners fully understand that the caregivers are not to be blamed for the happenings and a such offer them the needed support. Support is mainly emotional. Some of the caregivers shared their experiences as:

*“My family has been very supportive as well as my other children. My husband is educated, so he is fully aware it is no fault of mine, and hence I cannot be blamed for having a child with a disability.” (P2, mother, 35 years)*.

*“My husband has been helpful. He is a pastor, so he encourages me a lot. When I’m sad, down and feel very worried, he advises and encourages me not to worry. He tells me that worrying will not change the situation and that we should leave everything to God.” (P10, mother, 29 years)*.

### Sub-theme: maladaptive coping

Shifting blame was identified as a way of dealing with stress and making some caregivers feel better. They either blamed a supreme being for failing her after many years of trust. A caregiver mentioned:

*“As hard as I try, there are moments when I question God about what is happening in my life and to my child. Why did he have to let it happen to me? Especially when over the years I trusted him so much.” (P8, mother, 34 years)*.

Caregivers believed negligence and lack of prompt response from health workers caused the negative outcome on their children. Issues surrounding delivery and post-delivery care were cited as the cause of the DD. A caregiver shared her experience as:

*“My brother is outside the country and has explained to me what really causes such conditions. He told me that when there is a delay in delivery or if the child is pulled wrongly during delivery, such conditions can develop. When I went to deliver, it took so long for the baby to be delivered, and when the baby came, she was blue and needed special care. And so, when I reflect on such issues, I blame the midwives and doctors for my child’s condition.” (P10, mother, 29 years)*.

Caregivers blamed themselves for getting pregnant at an old age. They believed there was a connection between old age and giving birth to a child with DD. A caregiver illustrated:

*“Sometimes, I blame myself. I was told some of these conditions occur when you give birth in your old age. At the time I became pregnant, I was 44 years old. Maybe if I had not been pregnant in the first instance, the child would not go through this.” (P13, mother, 44 years)*.

## Discussion

The current study explored the experiences of caregivers’ children with DD using SPM as a framework. The use of the SPM did not limit the findings of the current study. Though the foundation of qualitative work is rich, detailed descriptions, but in a highly contextualised case, a weak framework may cause the details to become a story that is hard to adapt to different contexts [44]. The use of SPM helped make sense of complex social interactions and phenomena and facilitated a more explicit sense-making process. The experiences and coping mechanisms of mothers and fathers did not differ in the current study.

Participants in the current study were mostly females and reported that caregiving was stressful. The finding of the current study confirms another study which reported that being a female caregiver is linked with high levels of stress [55]. Also, stressful caregiving experiences are linked with low income levels [56] and most caregivers were unemployed or underemployed. Additionally, being married has been reported to buffer the stressful caregiving experience by providing certain resources [57] but half of caregivers were not married or had strained marital relationships.

### Perception of the caregiving role

Caregivers in the current study reported that caregiving was a highly stressful experience. One of the primary stressors in the SPM is the burdensome nature of caregiving. The diagnosis of DD alone has been identified as a significant predictor of stress among caregivers [58]. This may affect the caregiver as well as how they handle their children with DD. Parents experiencing high levels of stress may respond in several negative ways, including being less responsive towards the child, exhibiting an authoritarian style of parenting, and exhibiting neglectful behaviour [59]. Other outcomes of stress include being inconsistent with the disciplining of a child with a disability, having unrealistic expectations for the child with DD, and poor guidance [60]. The SPM affirms that, stressful caregiving experiences may lead to negative mental health outcomes for caregivers.

Caregivers stated that caring for a child with DD was time consuming. The National Alliance for Caregiving in collaboration with American Association of Retired Persons (AARP), reported that comparing the intensity of caregiving among children with special needs and adults with special needs, the former was quite intense and this was attributed to the number of hours involved in providing care [61]. It is estimated that the average number of hours spent providing care for a child with special needs was 29.7 h per week, which is more than the average 18.9 h per week spent caring for adults with special needs. The difference amounts to 11 h per week [61]. A systematic review assessing the daily patterns of time used by parents of children with complex needs revealed that these parents spent considerable time performing healthcare-related tasks beyond the normal parenting role [62]. This increased time commitment affects their ability to perform other tasks, including work, leisure activities and personal care [62]. As a result, most caregivers end up giving up on employment opportunities because they find it difficult to balance work with caregiving [62].

### Stressors associated with caregiving

In the context of the SPM, the current study identified both primary and secondary stressors. Primary stressors included the ADL needs of the child, communication barrier, managing challenging behaviours, child’s health needs and unmet educational needs. The secondary stressor identified was economic burden. Caregivers mentioned that assisting their children with ADLs was a source of stress, consistent with the SPM, which identifies ADL ability of the child with DD as a primary stressor. For example, Maridal et al. reported a correlation between psychological distress and performance of feeding ADL in caregivers of children with neurodevelopmental disorders [32].

Challenging behaviour exhibited by the children was identified as a main source of stress by caregivers of children with DD. The SPM and other studies recognize challenging behaviour as a primary stressor [63–65] and is linked to depression in caregivers [66, 67], distress [68], poorer family functioning [69], caregiver self-reported physical health problems [36, 70], fatigue [40], caregiving burden [20, 71], and poor immune responses in caregivers [72]. Behaviour problems in children have been categorized as internalising and externalising behaviours [73]. In the current study, caregivers predominantly mentioned externalising behaviours such as hyperactivity, poor impulse control, and aggression [73] as the sources of stress. Some studies have argued that the exhibition of a dominant dimension of behaviour problems, is influenced by the developmental stage of the child [73] with externalising behaviour more common in younger children and internalising behaviours in adolescents [74]. In the current study, mean age of children with DD was 6.28 years, possibly explaining why externalising behaviours were mainly mentioned by the caregivers.

Caregivers identified challenges with communication as a stressor. Communication skills of a child with DD has been strongly linked to the levels of caregiver stress [75]. The current study reported that poor communication skills led to high levels of stress and vice versa. Similarly, [76] reported that there was a statistically significant relationship between communication skills and caregiver wellbeing. Comparing children with DD to typically developing children, children with DD exhibit some delay in their language development, articulation, and fluency [76]. This makes their level of communication very limited because of their inability to comprehend language.

Caregivers understood the health needs of their children with DD; therefore, they did not underestimate the need for frequent medical assessment and care. Frequent visits to the hospital was identified as a source of stress. A systematic review indicated that children with DD were more likely to visit the emergency department compared to children without DD [77]. Similarly, [78] posited that children with intellectual and developmental disabilities use inpatient and emergency department services care at 1.8 times the rate of the general population. Frequent hospital visits disrupt caregivers’ schedules of activities and create additional stress, especially when healthcare facilities are far from home. A study shows positive relationship between caregiver stress and living more than 25 km from a healthcare facility or more from the caregiver’s residence to the health facility has been reported [79]. The distance created transportation difficulties for the caregivers in transporting their children to a health facility for care. Caregivers living in under-resourced or more distant areas from the health facility could attribute these issues to delaying or missing the child’s appointment [80]. During the extended journey to a healthcare facility, behavioural issues could intensify, leading to the child becoming increasingly uncooperative, thereby limiting the value of the visits [81]. Consequently, the child’s condition, cognitive functioning, and social interaction may not able to be improve and/or could even worsen.

Additionally, caregivers were worried about the education of their children. They reported difficulty finding and maintaining appropriate schools for their children with DD. This challenge of unmet educational needs for children with DD, is similar across low-, middle- and high-income countries. Tilahun et al. found that the majority of caregivers (74.5%) mentioned that their children did not have an appropriate educational provision [82]. Ambikile and Outwater reported that caregivers had social challenges pertaining to the child’s education as a result of the inadequate number of schools [83]. In their study, caregivers, had to spend a lot of time in trying to secure a school that could take their children. Consistent with the above studies, [84] reported that scarcity of educational activities as one of the main stressors for caregivers. This is known to impair the quality of life of the caregiver. Every parent would like their children to have access to education so they can become independent. Thus, not having access may be quite stressful for the caregivers.

Economic burden emerged as a significant stressor caregivers. They reported that the high cots associated with medication, diagnostic procedures and other related treatments had a negative impact on the finances. Consistent with the findings above, [85] and [86] reported that female caregivers experienced economic hardships as a result of increased expenses involved in health and transport, loss of their jobs as caregivers and a lack of savings. Caregiving had a negative effect on caregivers’ finances [32] because they were mostly housebound and had limited opportunities for working outside the home [87]. In many LMICs like Ghana, there is usually little or no governmental support which could lead to increased poverty. Consequently,  this situation could affect not only the caregiver but also the entire family. Studies have found a strong correlation between disability and poverty. The presence of disability heightens poverty risk and vice versa [88].

Caregivers reported that caregiving responsibilities often led to an inability to work, job loss, decreased work output and frequent excuses from work. The SPM identifies job strain as a secondary stressor that could affect the caregiver’s health negatively. Stabile and Allin reported described three pathways of economic cost related to caregiving: direct costs (related to the child’s disability), indirect costs (related to family coping strategies), and long-term costs (related to child’s future economic performance) [87]. Caregivers with children with disabilities frequently reduce their working hours or stop working altogether to meet caregiving demands[84, 87, 89] and this is consistent with the findings of the current study. This could be explained by the fact that the caregiving demands are often overwhelming coupled with inadequate social support which may lead caregivers to make such decisions about work. In the current study, few participants were employed in the formal sector, while most were involved in the informal sector. Regardless, caregiving had made some caregivers decide to quit their jobs because their time was diverted into caregiving such that they could no longer support their businesses. Others, too found that the hours involved in caregiving did not allow them to effectively combine or continue with their jobs and had to quit. Brehaut and colleagues reported that caregivers of children with disabilities were more likely to be unemployed [90]. In the this study, most caregivers were unemployed (8 out of 14).

### Negative health outcomes

Caregivers in the current study reported experiencing low back pain, headache and general body pains. This aligns with findings from other studies that have highlighted low back pain [28], generalised body aches [41]. Factors that have been associated with pain-related effects in caregivers include assisting with transfer the of children, caregivers mood and history of pain effects [91], dealing with problem behaviours and levels of difficulty in performing daily activities [28], regular carrying of the children [41]. Pain-related effects are something to be worried about because the caregivers may resort to poor coping mechanisms. For example, some caregivers in the current study resorted to regular abuse of analgesics which may have negative health consequences on their health. Again, increased pain related symptoms could interfere with the amount of available time needed to provide care for the child with DD.

In the current study, caregivers reported having interrupted sleep, restlessness at night and frequent awakenings. Consistent with the above, other studies have documented similar sleep problems among caregivers including lack of sleep, altered sleep patterns, increased wakefulness after sleep, and reduced sleep duration [38, 92, 93]. Caregivers in the current study attributed the sleep changes to worrying and poor sleep patterns in their children with DD. Similarly, [94] reported that changes in the sleep patterns of children with DD may negatively influence the sleep patterns and daytime functioning of other family members. Poor sleep quality has been linked to increased levels of stress, anxiety and depression [95].

Psychologically, caregivers reported a lack of concentration, anger, shifting blame and feelings of sadness. The SPM identifies changes in the psychological well-being of the caregiver as outcomes of caregiving. In line with the above study, a qualitative study examining the burden of caregivers of children with CP, reported that mothers experienced guilt, blame, and worry in relation to the future of the child with DD disability [96]. In a qualitative study, Asa et al. reported caregivers feeling frustrated, sad, angry, worried, inferior due to the rejection of their children by other children without disability [86]. Caregivers in the current study reported that they were anxious about the future. Gomez et al. reported in a literature review that caregivers had concerns about the children’s future, attributed to the caregivers identifying that they had limitations in providing support for them [97]. In their systematic review, [15] posited that one of the themes identified was caregivers worries about the future. Other studies have reported the same [98, 99]. It is possible caregivers of the current study had little or no emotional support, as many mentioned feelings socially isolated and having strained social relationships.

In the current study, caregivers reported social isolation mainly due to the difficulty of managing the child with DD in public and negative attitudes from the public. Consistent with the above, [96] reported that caregivers felt a sense of isolation in society. Mkabile et al. further explain that caregivers felt ashamed about their child’s condition, which influenced their inability to take them out, thus hiding them from the community to avoid discriminating and stigmatising them [15]. Lamptey reported that parents had difficulty managing behaviour challenges in children with IDD in public [100], leading to social isolation. Though related but not the same concept, other studies have found a relationship between informal caregiving and loneliness [101, 102]. Loneliness refers to the feeling of poor quality or size of the caregiver’s social network while social isolation is the perception of the caregiver that he or she does not belong to society. In terms of relationships, caregivers reported strained relationships with partners, family, friends, and society at large, which means that their social network size has reduced leading to a feeling that they are no longer needed in society. The SPM identifies family strains as a secondary stressor meaning they could lead to negative physical and mental health outcomes. Consistent with the current study, the findings by [86] showed that caregivers experienced reduced social interaction. Caregivers explained it was a result of fathers not accepting their children with disabilities, increased time spent providing care for the child and stigmatisation and discrimination towards their children with disability.

### Coping strategies

Caregivers used both adaptive and maladaptive coping strategies in caring for their children. The caregivers identified that they used prayers to cope. A review of the literature indicates that one of the main mechanisms used in coping with stress associated with caring for a child with DD is the use of spirituality [82, 103, 104]. Asa et al. in a qualitative study reported that all participants in their study mentioned that they prayed to God [105]. Resorting to religious coping has been identified as an effective way of coping with stress [106]. The exact mechanism by which it works cannot be determined, however, it may be because it helps in accepting the current situation and accepting to deal with it according to God’s will [15]. Additionally, religious belief may produce endurance and resistance in individual’s dealing with stress [106]. Engaging in religious coping is likely to lead to being calm, feeling peaceful and being less stressful. While some studies support the idea that religious coping may reduce stress and other mental health outcomes, other studies did not find such a relationship both in the short term [107] and the long term (Lyons et al., 2010).

Some caregivers in the current study mentioned that they depended on themselves to cope with the stress associated with caregiving. Asa et al. reported that caregivers used self-reliance to cope with challenges in caring for children with disabilities [105]. They report that caregivers relied on their own capabilities. In the current study, caregivers sought support from family and friends. It was emotional support and they believed it helped them to deal with the stress involved in caregiving. Consistent with the above, [106] identified seeking support (37.8%) as the second most useful coping resource. The findings of the current study indicate that some caregivers use maladaptive coping strategies to deal with stress. Specifically, the use of blame was quite significant. Studies have indicated that blame is used as a defense mechanism to avoid feelings of guilt, sadness, powerlessness, and shame [108].

### Limitations of the study

There are some limitations to the current study that are typical of qualitative research.The translation of the interview transcripts was done from Twi to English, this carries inherent limitations that need to be acknowledged. Some words in Twi do not have direct translation in English; in some cases, words near in English meaning are used or adopted to (re)present participants’ experiences. Our findings might not apply to all caregivers of children with disabilities due to potential bias selection. A few caregivers who possessed significant insights might not be attending the Neurodevelopmental clinic at the KBTH, which would have prevented them from taking part. Perceptions may differ among caregivers of children with disabling conditions that were not included in this sample, despite the fact that the children of the caregivers in this study had a wide range of diagnoses.

### Recommendations

Based on the findings of the current study, the authors recommend that:

First and foremost, programmes aimed at helping caregivers change their perception should be instituted. Negative perception of the caregiving roles and process could impact negatively the health and well-being of caregivers. Programmes that could help the caregivers perceive their caregiving role more positively should be implemented Ministry of Health and Ghana Health Service.

Second, stress management programmes should be instituted for caregivers of children with DD. The programmes should compromise those that they can do without support at home such that days during which they do not bring their children to the hospital, they can still practice at home.

Third, the Ministry of Health together with other stakeholders should implement a national Caregiver Policy, wherein, under the policy, caregivers of children with DD will undergo regular screening assessing physical, psychological and social health. The screening could be incorporated into the regular clinic visits of the child with DD. Caregivers who perform poorly on the screening tool may then be given further assessment and support.

Lastly, a support group should be created, wherein caregivers are given the opportunity to share their experiences and coping resources. This could be a strong buffering system for caregivers. A different meeting day could be problematic, as caregivers already have full schedule associated with their caregiving role. Thus, these support groups could be incorporated into the normal clinic attendance.

## Conclusion

The findings of the current study as mirrored in the context of the SPM highlights that caregivers have varied caregiving experiences and most express negative consequences mainly affecting their physical, psychological and social well-being. Thus, the health of caregivers should be prioritized as a caregiver who is healthy may translate into providing proper support for the child with DD, subsequently, improving the well-being and overall quality of life of the child with DD. The SPM emphasizes on how various factors are linked in providing negative health outcomes in the caregiver, a comprehensive approach which considers how these factors are linked, should be used in addressing the caregiver’s challenges.

### Electronic supplementary material

Below is the link to the electronic supplementary material.

Supplementary Material 1

## Data Availability

The authors confirm that the data supporting the findings of this study are available within the article and its supplementary materials.
