# Supplementary material for: Caring by default: experiences of caregivers of children with developmental disabilities in Ghana mirrored in the context of the stress process model
Source: BMC Nurs. 2024 Jul 15;23:482. doi: 10.1186/s12912-024-02142-1 (PMC11251246; doi:10.1186/s12912-024-02142-1)
Supplement: Supplementary file 1 — Supplementary Material 1 [file 12912_2024_2142_MOESM1_ESM.docx]

**Semi-structured interview guide**

**Opening**

Establish rapport

Outline purpose

Justification

Duration of interview

**Background/demographics**

| Gender |  | Employment: |  |
| --- | --- | --- | --- |
| Age |  | Number of children |  |
| Level of education |  | Relationship to child with DD |  |
| Marital status |  | Duration of care |  |
| Number of caring hours per day |  |  |  |

**Question (to follow-up each question with probes where necessary)**

1. Please tell me about your experience in caring for your child with DD.

Probes:

Tell me how you came to be involved in caring for the child with DD.

Describe a typical day in caring for a child with DD at home.

Tell me about how you feel about your role as a caregiver for a child with DD.

1. Describe the needs of the child with DD that makes caregiving difficult for you?

Probes:

Is the child able to perform activities of daily living for himself/herself?

Are you able to communicate your needs to your child with DD?

Is your child with DD able to communicate his needs to you?

Is your child receiving education just like any other child?

1. In which ways have caring for a child with DD affected your life?

**Probes:**

How has caring for a child with DD affected your physical health?

How does caring for a child with DD affect you psychologically?

How has your social wellbeing and relationships been affected by caring for a child with DD?

Has caring for your child affected your finances?

1. How do you cope with caregiving?

**Probes:**

Can you tell me the specific things you do to deal with your caregiving role?

**Closure**

Summarize

Maintain rapport
